# Characteristics of brain glucose metabolism in Parkinson’s disease patients with freezing of gait: a study based on ^18^F-FDG PET imaging and deep learning

**DOI:** 10.1186/s12883-025-04468-y

**Published:** 2025-10-31

**Authors:** Zhuang Zhu, Yao Geng, Xixi Wang, Jiaxin Shi, Hualin Wang, Linghui Liu, Shengrong Li, Caiting Gan, Yongsheng Yuan, Qi Zhu, Kezhong Zhang

**Affiliations:** 1 Department of Neurology, The First Affiliated Hospital with Nanjing Medical University, Nanjing, China; 2 College of Computer Science and Technology, Nanjing University of Aeronautics and Astronautics, Nanjing, China; 3 Department of Neurology, Nanjing First Hospital, Nanjing Medical University, Nanjing, China

**Keywords:** Parkinson’s disease, Freezing of gait, ^18^F-FDG PET, Brain glucose metabolism, 3D convolutional neural network

## Abstract

**Objective:**

Freezing of gait (FOG) is a common gait disorder in the advanced stages of Parkinson’s disease (PD), closely associated with impaired balance and executive function. This study aimed to investigate specific changes in brain glucose metabolism in FOG patients using ^18^F-FDG PET. Deep learning methods were utilized to offer valuable perspectives for identifying FOG.

**Methods:**

Eighteen PD patients with FOG(PD-FOG), 11 patients without FOG (PD-NFOG) and 17 healthy controls (HC) were recruited. All participants underwent ^18^F-FDG PET imaging, and group comparisons were employed, to identify regions with significant differences in glucose metabolism. 3D convolutional neural network (3D CNN), as well as traditional machine learning models, were constructed for the automatic identification of the FOG type.

**Results:**

PET imaging analysis showed that the differences between the PD-FOG group and the PD-NFOG group were mainly located in the frontal lobe, parietal lobe and cingulate gyrus. The 3D CNN achieved diagnostic accuracies of 90.09% for distinguishing PD and 95.40% for FOG, surpassing other machine learning models. The 3D CNN achieved the smallest mean squared error (MSE), amounting to 48.01, in the prediction of Freezing of Gait Questionnaire (FOG-Q) scores.

**Conclusion:**

Specific glucose metabolism patterns in PD-FOG mainly covered the frontoparietal network (FPN). The integration of ^18^F-FDG PET imaging with deep learning methods effectively differentiated patients with FOG. The 3D CNN exhibited a high diagnosis accuracy level, providing reliable imaging and artificial intelligence support for PD with FOG.

**Supplementary Information:**

The online version contains supplementary material available at 10.1186/s12883-025-04468-y.

## Introduction

Parkinson’s disease (PD) is a common neurodegenerative disorder of the central nervous system, characterized by degeneration of dopaminergic neurons in the substantia nigra and the formation of Lewy bodies [1]. Freezing of gait (FOG), characterized by brief episodes where the patient is unable to initiate steps, is a frequent gait disorder in the later stages of PD [2]. FOG can result in a heightened likelihood of falls and a substantial deterioration in patients’ quality of life. Prompt and precise diagnosis of FOG, along with an accurate assessment of its severity, are crucial for effective treatment and management. Currently, clinicians primarily rely on clinical scales such as the motor section of the Unified Parkinson’s Disease Rating Scale (UPDRS), the Freezing of Gait Questionnaire (FOG-Q), and gait rating scales to diagnose FOG. These scales assess the severity of FOG by subjectively scoring the patient’s gait, motor symptoms, and the frequency and duration of freezing. However, these methods depend heavily on physician observation and patient self-reports, which can introduce subjectivity and bias, and may fail to detect minor, fleeting freezing events. Although clinicians can also use the gait analysis systems to objectively evaluate the gait characteristics, such as recording step frequency, step length, gait symmetry, etc. Despite the advantages of gait analysis, there are still limitations in capturing the symptoms of FOG. Especially for transient and irregular episodes, the device resolution and the analysis accuracy would restrict the early diagnosis and continuous monitoring of FOG.

The application of ^18^F-fluorodeoxyglucose positron emission tomography (^18^F-FDG PET) in neurodegenerative diseases, especially PD, has been widely studied and has shown important clinical value. Compared with other imaging techniques, such as magnetic resonance imaging (MRI) or computed tomography (CT), ^18^F-FDG PET can provide valuable information for evaluating brain function by directly measuring glucose metabolism in brain regions, especially subtle changes in metabolic activity [3, 4]. In diagnosing PD, ^18^F-FDG PET helps assess the presence and severity of the disease by monitoring changes in glucose metabolism in key brain regions such as the basal ganglia, cortex, and thalamus [5]. In addition, ^18^F-FDG PET can also help to differentiate different types of parkinsonism (e.g., PD and multiple system atrophy), which have different metabolic features in the brain [6, 7]. The occurrence of FOG is closely related to the dysfunction of multiple key brain regions, especially those responsible for motor control and cognitive regulation, including basal ganglia, prefrontal cortex and parietal cortex. Studies have shown that metabolic abnormalities in these brain regions are one of the important pathological features of FOG [8]. However, the mechanism of FOG is not the dysfunction of a single brain region but a complex dynamic network imbalance between multiple brain regions. Studies have shown that FOG patients have abnormalities in the frontal-basal ganglia network, cortex-cerebellar pathway and cortex-cortical connectivity [9–11]. These network imbalances may be a key reason for the impaired signal transmission between higher-order cognitive functions and motor execution, especially when patients are coping with complex tasks such as turning or obstacle avoidance, and insufficient network regulation can exacerbate the occurrence of FOG [12–14]. Therefore, ^18^F-FDG PET is expected to become an effective complementary tool to identify and evaluate the symptoms of FOG and provide a new research perspective for exploring the pathological mechanism of FOG.

In recent years, deep learning-based models, especially convolutional neural networks (CNNs), have been increasingly used in neuroimaging [15]. Nuclear medicine may benefit more from advanced deep learning applications than brain magnetic resonance imaging [16]. Previous studies have primarily focused on the application of 2D CNNs, while relatively few studies have explored the use of 3D CNNs in FOG [17, 18]. Compared with other traditional deep learning methods (such as 2D CNNs, GCNs, etc.), 3D CNNs have the following advantages: they are capable of capturing spatial and spatiotemporal features in 3D data, automatically extracting high-level spatial features, reducing human intervention, and being highly adaptable, able to process large amounts of PET data [19, 20]. When analyzing ^18^F-FDG PET data, 3D CNNs can perform classification, detection, localization, segmentation, quantification, and radiomics feature extraction, thereby capturing comprehensive multidimensional changes in patients’ brain metabolism, significantly improving the recognition ability of FOG, enhancing diagnostic sensitivity and accuracy, reducing human errors, and improving result consistency [21].

Based on this, ^18^F-FDG PET imaging was used to explore the changes of brain metabolic patterns in PD-FOG patients. In addition, a PD-FOG model based on 3D CNN was constructed and compared with six other traditional models to evaluate their capabilities in identifying PD-FOG, which might provide new insight into the pathophysiological mechanisms underlying FOG.

## Subjects and methods

### Subjects

This study enrolled 29 patients with idiopathic Parkinson’s disease (PD) and 17 healthy controls who visited the Department of Neurology at the First Affiliated Hospital of Nanjing Medical University between June 2021 and October 2024. All participants provided written informed consent, and the study protocol was approved by the Ethics Committee of the First Affiliated Hospital of Nanjing Medical University, in strict compliance with the principles of the Declaration of Helsinki.

#### Study groups

Participants were divided into three groups: (1) Healthy control group (*n* = 17): Demographically matched to the PD group, with no neurological abnormalities. (2) PD with freezing of gait group (PD-FOG group, *n* = 18): Met diagnostic criteria for both PD and FOG. (3) PD without freezing of gait group (PD-NFOG group, *n* = 11): Met PD diagnostic criteria but exhibited no FOG manifestations.

#### PD patient inclusion criteria

(1) Diagnosed with idiopathic PD according to the International Movement Disorder Society clinical diagnostic criteria [22]; (2) Aged 40–80 years; (3) Absence of severe tremor or levodopa-induced dyskinesia; (4) Ability to walk independently for ≥ 30 m; (5) Stable medication regimen for ≥ 3 months.

#### FOG diagnostic criteria (required concurrent PD diagnosis) [23]

(1) FOG-Q item 3 score ≥ 2;(2) Observation of typical freezing phenomena (e.g., gait initiation delay, abrupt step shortening, or complete arrest) during standardized provocation tests; (3) At least one clinically documented FOG episode during evaluation [23].

#### Healthy control inclusion criteria

(1) Matched to the PD group in age, sex, and years of education; (2) No history or clinical signs of neurological disorders; (3) Independent walking ability ≥ 30 m; (4) FOG-Q item 3 score = 0 and negative provocation test results.

#### Exclusion criteria (applied to all participants)

(1) Comorbid neurological disorders (e.g., stroke, multiple sclerosis); (2) Contraindications to PET/CT imaging; (3) Musculoskeletal disorders, uncorrected visual impairment, or psychiatric conditions; (4) Imaging-confirmed focal brain lesions; (5) Severe resting tremor; (6) Cognitive impairment (MoCA < 26) [24]; (7) Participation in studies affecting gait or cognition within the preceding 3 months.

### Methods

#### Data collection and clinical assessments

Demographic information, including age, sex, years of education, disease duration and levodopa equivalent daily dose (LEDD) were collected. The modified Hoehn-Yahr (mH-Y) stage [25] and Unified Parkinson’s Disease Rating Scale (MDS-UPDRS) [26] were used to systematically assess the clinical severity of PD. Tinetti activity test and Timed Up and Go (TUG) were used to assess the severity of balance and gait symptoms. The FOG-Q was obtained to quantify the severity of FOG. The Montreal Cognitive Assessment (MoCA) [24], Frontal assessment Battery (FAB) [27], Epworth Sleepiness Scale (ESS) [28] and Apathy Scale [29] were used to assess cognitive function, executive function, sleepiness, and global affective symptoms. All clinical assessments were performed at least 12 h after discontinuation of antiparkinsonian drugs.

#### PET imaging protocol

^18^F-FDG PET examinations were performed in 3D mode using a Siemens Biograph 16 HR PET/CT scanner in a quiet, dimly lit room in the PET Center of the First Affiliated Hospital of Nanjing Medical University. All subjects underwent FDG-PET imaging in the morning after overnight fasting and withdrawal of dopaminergic drugs, with confirmed blood glucose levels below 7.0 mmol/L. Following intravenous administration of ^18^F-FDG (dose range: 3.7–5.5 MBq/kg), participants rested in a quiet, dimly lit room for a 60-minute uptake period. During this period, conditions were controlled: participants were instructed to remain awake, with their eyes open and ears unoccluded, in a supine position, and to minimize movement and speech. After the uptake period, a 10-minute static emission scan was acquired in 3D mode. The subject was positioned along the orbitomeatal line (OML) and, to correct for photon attenuation, a low-dose CT transmission scan was performed immediately prior to the emission scan. Emission data were reconstructed using the ordered subset expectation maximization (OSEM) algorithm. Using scanner-specific filters, PET data were corrected for attenuation and scattered radiation and reconstructed into a volumetric image with a 256 × 256 matrix and 1.22 mm voxel size. A single static image per subject, representing the accumulated activity from approximately 60–70 min post-injection, was generated and used for all subsequent analyses. No dynamic scanning or DVR images were generated in this study.

#### PET image transformation and statistical analysis

In this study, FDG-PET data analysis was performed using SPM12, implemented in Matlab 7.9 (MathWorks, Sherborn, MA, USA). The linear and nonlinear transformations through which participants scanned first were normalized to the Montreal Neurological Institute (MNI) standard stereotactic space [30]. All FDG-PET images were intensity-normalized to the global mean brain uptake, which is a widely used semi-quantitative approach in clinical FDG-PET group analyses when arterial input function is not available. This semi-quantitative global mean normalization approach is widely adopted in clinical FDG-PET group analyses when arterial input function is unavailable, and ensures comparability across subjects. The images were then smoothed with a 10-mm Gaussian filter to improve the signal-to-noise ratio and to enhance the detection of subtle anatomical variations. Statistical comparisons between groups were conducted entirely within SPM12. A voxel-level threshold of *p* < 0.001 (uncorrected) with a cluster extent threshold of 25 voxels was applied, using an explicit gray matter mask to restrict the analysis. The resulting statistical maps, from which the Gaussianized t-values (reported as Z values in Table 2, representing statistical significance) were derived, were then used for subsequent region of interest (ROI) analysis. The MarsBaR toolbox (version 0.44) was used solely for ROI definition and value extraction. The thresholded statistical maps from SPM were imported into MarsBaR. The mean metabolic value within each ROI was then extracted for every subject. These values were used to assess the relative glucose metabolism in each significant brain region. In order to further explore the independent correlation between the severity of freezing gait and the difference of FDG metabolism in brain regions between the PD-FOG group and the PD-NFOG group, Partial Correlation Analysis was performed with the help of SPSS software (version 26.0). The freezing of gait score and FDG metabolism in different brain regions were set as the key variables in the analysis, and the confounding factors reflecting the severity of disease progression, including UPDRS-Ⅲ and LEDD, were controlled. In this way, the interference caused by confounding variables was eliminated, and the partial correlation coefficient between the severity of freezing of gait and FDG metabolism value in different brain regions was accurately calculated, in order to evaluate the true correlation between the differential brain regions and freezing of gait score more objectively and accurately. The significance of partial correlation coefficient was determined by two-tailed t test, and the significance threshold was set as α = 0.05, and the statistical results were corrected by False Discovery Rate (FDR).

#### Proposed deep learning based Parkinson prediction model

**Fig. 1:**
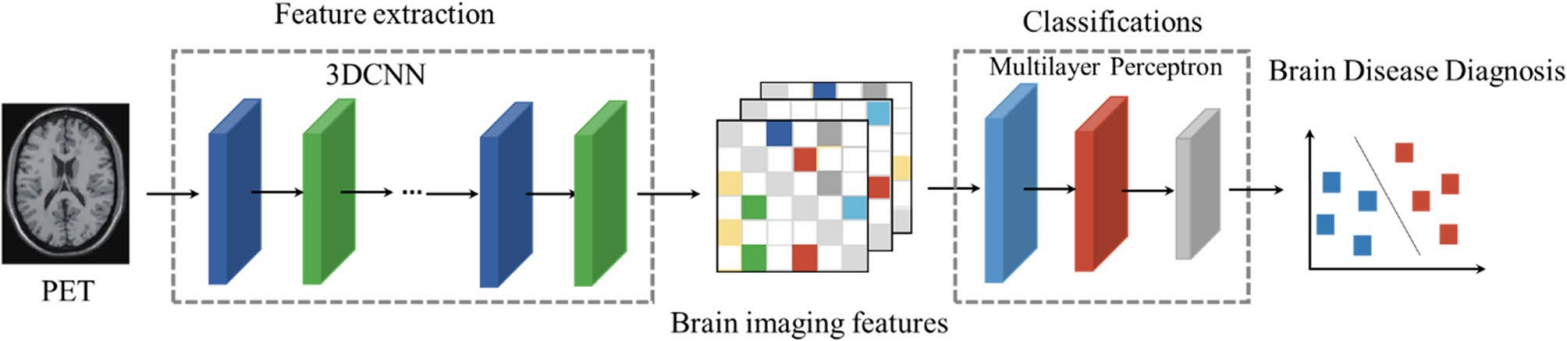
The workflow of the 3D CNN model

We constructed a specialized 3D CNN prediction model (as shown in Fig. 1, with the deep learning model code provided in Appendix 1), which implements a three-dimensional convolutional neural network for 3D brain imaging data classification. The network consisted of three 3D convolutional layers and pooling layers, designed to gradually extract features and reduce spatial dimensions. Each convolutional layer was followed by a max-pooling layer with a 2 × 2 × 2 window and a stride of 2. The output channel numbers of the convolutional layers were 16, 32, and 64, respectively, with a kernel size of 3 × 3 × 3, and ReLU activation functions were applied at each layer. After the convolution and pooling operations, the feature maps were flattened into a one-dimensional vector and fed into two fully connected layers. The first fully connected layer reduced the feature dimension to 128, and the second layer output predictions for two classes. The network’s output was passed through a LogSoftmax activation function to produce the logarithmic probabilities for each class. The negative log-likelihood loss function (F.nll_loss) was used to compute the difference between predictions and ground-truth labels, guiding the optimization of model parameters through the backpropagation algorithm. This loss function adjusted the model weights to minimize prediction errors, thereby improving classification accuracy. This network was well-suited for 3D data classification tasks, such as medical image analysis.

In addition to the proposed 3D CNN, we also implemented several traditional machine learning and deep learning models to provide comparative baselines. These included: Multi-Layer Perceptrons (MLPs) [31], Support Vector Machines (SVMs) [32], Graph Convolutional Neural Networks (GCNs) [33], tensor decomposition approaches such as Tucker [34] and CP decomposition [35], and 2D CNNs [36].

To ensure a comprehensive and unbiased evaluation, we adopted a 10-fold cross-validation (10-FCV) scheme. The entire dataset was first randomly shuffled, then partitioned into 10 non-overlapping folds of approximately equal size. In each iteration, one fold was reserved as the independent test set, while the remaining nine folds were used for model training and internal validation. Within these nine folds, 80% of the data was used for training, and 20% was used as a validation set to monitor performance and apply early stopping. The model achieving the highest validation accuracy within each fold was saved and subsequently evaluated on the corresponding independent test fold. This process was repeated 10 times, and each subject appeared once in the test set.

For ROC curve analysis, the prediction probabilities from all test folds were concatenated, along with their corresponding ground-truth labels, to generate a global ROC curve using the scikit-learn implementation. The area under the curve (AUC) was calculated to quantify classification performance. As ROC analysis is direction-invariant, when the scikit-learn implementation produced AUC values < 0.5 due to label inversion, we recalculated them as 1 – AUC to ensure all discriminatory abilities were expressed as ≥ 0.5.

The following hyperparameters were explicitly set for reproducibility: Optimizer: Adam; Learning rate: explored in the range {3 × 10^−4^,5 × 10^−4^,7 × 10^−4^,1 × 10^−3^,3 × 10^−3^,5 × 10^−3^}, with 3 × 10^−3^ as the final selected value; Weight decay: explored in {0,1 × 10^−4^,1 × 10^−3^,2 × 10^−3^}, with 2 × 10^−3^ as the final selected value; Batch size: 16 for training/validation, 1 for testing; Epochs: up to 300; Early stopping: training terminated if validation accuracy did not improve for 100 consecutive epochs; Loss function: negative log-likelihood loss (F.nll_loss).

Model performance was evaluated using accuracy, precision, recall, F1-score, and AUC, averaged across the 10 test folds. This rigorous evaluation ensured that the reported metrics reflect robust and generalizable model performance in predicting FOG in PD patients.

#### Statistical analysis

Statistical analysis was performed using SPSS 26.0 software (IBM, Armonk, NY, USA) for all statistical computations. The Shapiro–Wilk test was used to assess the normality of the distribution of all variables. Continuous variables were analyzed using one-way ANOVA for normally distributed data and the Kruskal-Wallis test for non-normally distributed data across multiple groups. Pairwise comparisons between two groups were conducted using the two-sample t-test for normally distributed data and the Mann-Whitney U test for non-normally distributed data. Categorical variables, such as gender distribution, were analyzed using Fisher’s Exact Test. Bonferroni correction was applied for multiple comparisons to control for Type I error. *P* < 0.05 was considered statistically significant. All continuous variables were expressed as mean ± standard deviation (SD), and categorical variables were presented as frequencies and percentages. Statistically significant differences were observed in MDS-UPDRS part III, LEDD, TUG, Tinetti balance subscale, and Tinetti gait subscale across groups, with detailed pairwise comparisons reported using Bonferroni correction.

## Results

2.1 A general clinical data comparison was shown in Table 1. There were no significant differences in age, gender, years of education, MoCA, FAB, ESS and Apathy among the three groups (PD-FOG group, PD-NFOG group and HC group). There was no significant difference in disease duration, age at onset, UPDRS Ⅰ, UPDRS II and mH-Y grade between the PD-FOG group and the PD-NFOG group. Compared with the PD-NFOG group, the PD-FOG group had significantly higher UPDRS III (*P* = 0.020), LEDD (*P* = 0.034), TUG (*P* = 0.005), and FOG-Q (*P* < 0.001). Tinetti balance subscale (*P* < 0.001) and Tinetti gait subscale (*P* < 0.001) were significantly lower. The scores TUG (*P* < 0.001) in the PD-FOG group were significantly higher than those in the HC group.

**Table 1:** Demographic and clinical characteristics of all subjects

Variable	PD(*n* = 29)				Post hoc (Bonferroni correction)
	PD-FOG(*n* = 18)	PD-NFOG(*n* = 11)	HC(*n* = 17)	*P* value	
Age (y)	64.17 ± 6.43	61.55 ± 7.81	62.87 ± 5.10	0.622^b^	
Gender (M/F)	11/7	6/5	8/7	0.928^a^	
Education (y)	12.06 ± 4.33	11.00 ± 2.24	10.07 ± 3.06	0.147^c^	
Disease duration (y)	3.29 ± 2.54	3.60 ± 3.81	-	0.735^d^	
Age at onset (y)	60.98 ± 6.81	57.95 ± 6.15	-	0.238^d^	
UPDRS part I	2.83 ± 2.38	3.36 ± 2.46	-	0.554^d^	
UPDRS part II	12.83 ± 3.02	10.36 ± 4.08	-	0.071	
UPDRS part III	21.72 ± 9.83	13.36 ± 8.30^d*^	-	0.020^d, *^	
mH-Y stage	2.36 ± 0.47	2.04 ± 0.52	-	0.106	
LEDD (mg)	321.39 ± 217.09	136.36 ± 181.35^d*^	-	0.034^d, *^	
MoCA	26.22 ± 1.23	26.77 ± 0.90	26.50 ± 0.85	0.273^c^	
FAB	16.35 ± 1.62	17 ± 1.5	17.23 ± 0.83	0.194^c^	
ESS	4.39 ± 3.79	4.11 ± 3.89	2.08 ± 1.44	0.215^c^	
Apathy	20 ± 10.38	15.78 ± 6.36	14.69 ± 6.03	0.276^c^	
TUG(s)	19.62 ± 6.97	12.15 ± 2.28	9.74 ± 0.77	< 0.001^c,***^	PD-FOG > PD-NFOG
					(*P* = 0.005^**^)
					PD-FOG > HC
					(*p* < 0.001^***^)
					PD-NFOG > HC
					(*p* = 0.019^*^)
Tinetti balance subscale	12.33 ± 3.22	16 ± 0	-	< 0.001^d,***^	
Tinetti gait subscale	7.94 ± 2.75	11.89 ± 0.33	-	< 0.001^d,***^	
FOG-Q	10.33 ± 6.63	-	-	< 0.001^d,***^	

Value were presented as the mean ± standard deviation. *p*<0.05 was considered statistically significant

*Abbreviations*: *PD* Parkinson’s Disease, *FOG* Freezing of gait, *HCs* Healthy controls, *M* Male, *F* Female. *y* year, *UPDRS* Unified Parkinson’s disease rating scale, *mH-Y* *stage* modified Hoehn and yahr rating scale, *LEDD* levodopa equivalent daily dose, *MoCA* Montreal Cognitive Assessment, *FAB* Frontal Assessment Battery, *ESS* Epworth Sleepiness Scale, *TUG* Timed Up and Go Test, *FOG-Q* Freezing of Gait Questionnaire

^a^Fisher’s Exact Test

^b^One-way ANOVA.

^c^Kruskal-Wallis.

^d^Mann-Whitney U

^*^P < 0.05

^**^P < 0.01

^***^P < 0.001

### PET data

**Fig. 2:**
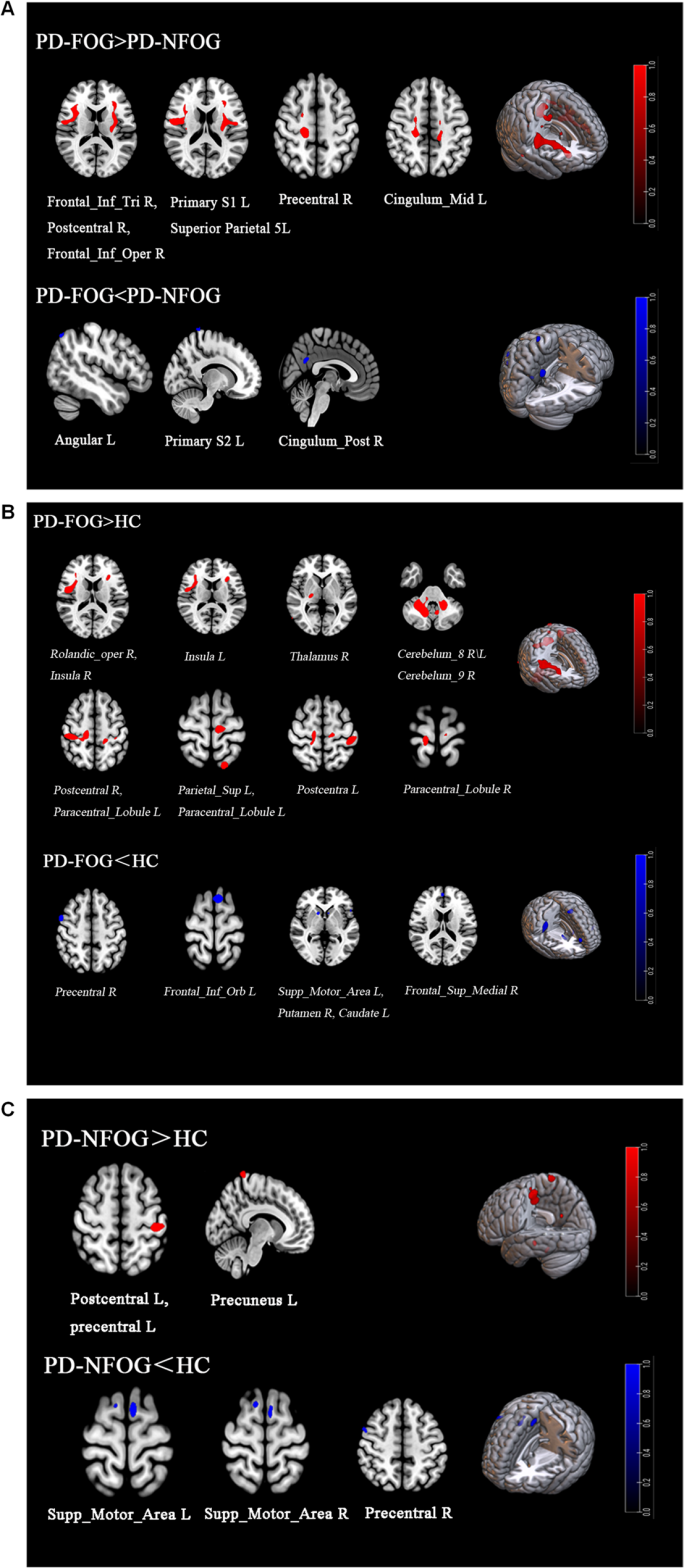
Differences in glucose metabolism among the PD-FOG, PD-NFOG, and HC groups. **A** Differences in glucose metabolism among the PD-FOG and PD-NFOG groups; **B** Differences in glucose metabolism among the PD-FOG and HC groups; **C** Differences in glucose metabolism among the PD-NFOG and HC groups

All reported metabolic differences were derived from voxel-based group analyses in SPM12, not ROI-based tests. Voxel-based analysis in SPM12 revealed that, compared with the PD-NFOG group, the PD-FOG group showed increased glucose metabolism in the right inferior triangular part of the frontal gyrus, the right postcentral gyrus, the right opercular part of the right inferior frontal gyrus, the left primary somatosensory area 1, the left superior parietal lobule 5 L area, the right precentral gyrus and the left middle cingulate gyrus. Glucose metabolism was decreased in the left angular gyrus, left primary somatosensory area 2, and right posterior cingulate gyrus (Table 2; Fig. 2A). Compared to the HC group, the PD-FOG group demonstrated increased glucose metabolism in the right Rolandic operculum, bilateral insula, bilateral eighth cerebellar lobule, right ninth cerebellar lobule, bilateral postcentral gyrus, left superior parietal gyrus, right thalamus, and bilateral paracentral lobule. Decreased glucose metabolism was observed in the right precentral gyrus, left supplementary motor area, left orbital part of the inferior frontal gyrus, right putamen, right superior medial frontal gyrus, and left caudate regions (Table 2; Fig. 2B). Furthermore, compared to the HC group, the PD-NFOG group showed increased metabolic activity in the left postcentral gyrus, left precentral gyrus, and left precuneus, while decreased metabolic activity was found in the bilateral supplementary motor area and right precentral gyrus regions (Table 2; Fig. 2C).

**Table 2:** Differences in glucose metabolism between brain regions^*^

Brain area (AAL,▲HCP-MMP1.0)	Side	Cluster size	MNI coordinate			Z score
			X	Y	Z	
PD-FOG > PD-NFOG						
Frontal_Inf_Tri	R	754	30	36	2	4.62
Postcentral	R	754	50	−6	18	4.16
Frontal_Inf_Oper	R	754	34	14	16	4.11
▲Primary Somatosensory 1	L	555	−28	26	16	4.20
▲Superior Parietal 5L	L	555	−34	−12	34	4.02
Precentral	R	373	16	−22	60	4.09
Precentral	R	373	20	−6	50	3.58
Cingulum_Mid	L	83	−18	−26	46	3.47
Cingulum_Mid	L	83	−16	−10	48	3.39
PD-FOG < PD-NFOG						
Angular	L	27	−48	−68	52	3.6
▲Primary Somatosensory 2	L	38	−14	−40	82	3.45
Cingulum_Post	R	60	4	−54	28	3.37
PD-FOG > HC						
Rolandic_oper	R	513	48	−2	16	4.54
Insula	R	513	34	28	12	3.82
Insula	R	513	32	16	14	3.72
Cerebellum_8	R	1764	26	−44	−40	4.46
Cerebellum_8	R	1764	16	−62	−50	4.37
Cerebellum_8	R	1764	14	−60	−36	3.91
Insula	L	102	−30	24	14	4.15
Cerebellum_9	R	1052	20	−46	−40	4.03
Cerebellum_8	R	1052	14	−68	−50	3.55
Cerebellum_8	L	1052	−8	−60	−32	3.31
Postcentral	R	1052	38	−28	52	3.61
Postcentral	R	1052	20	−34	58	3.51
Parietal_Sup	L	61	−18	−66	68	3.85
Thalamus	R	87	22	−18	4	3.66
Paracentral_Lobule	L	141	−10	−24	70	3.62
Postcentral	L	145	−44	−30	62	3.59
Paracentral_Lobule	L	62	−18	−34	54	3.56
Paracentral_Lobule	R	37	12	−30	76	3.5
PD-FOG < HC						
Precentral	R	143	52	2	54	4.5
Precentral	R	143	62	6	42	3.72
Supp_Motor_Area	L	82	−8	8	70	3.8
Frontal_Inf_Orb	L	34	−54	20	−2	3.61
Putamen	R	74	14	12	2	3.44
Frontal_Sup_Medial	R	49	2	52	18	3.34
Caudate	L	32	−8	12	2	3.25
PD-NFOG > HC						
Postcentral	L	332	−48	−30	66	4.41
Postcentral	L	332	−34	−28	50	4
Precentral	L	332	−36	−22	68	3.22
Precuneus	L	73	−10	−46	78	3.98
PD-NFOG < HC						
Supp_Motor_Area	L	51	−6	0	70	3.58
Supp_Motor_Area	R	38	10	10	66	3.45
Supp_Motor_Area	R	38	10	2	72	3.25
Precentral	R	33	50	2	52	3.35

*Abbreviations*: *Frontal_Inf_Tri* Inferior Frontal Gyrus, triangular part, *Frontal_Inf_Oper* Inferior Frontal Gyrus, opercular part, *Cingulum_Mid* Middle Cingulate Gyrus, *Cingulum_Post* Posterior Cingulate Gyrus, *Rolandic_oper* Rolandic Operculum, *Cerebellum_8* Cerebellum Lobule VIII, *Cerebellum_9* Cerebellum Lobule IX, *Parietal_Sup* Superior Parietal Lobule, *Paracentral_Lobule* Paracentral Lobule, *Supp_Motor_Area* Supplementary Motor Area, *Frontal_Inf_Orb* Inferior Frontal Gyrus, orbital part, *Frontal_Sup_Medial* Superior Medial Frontal Gyrus, *AAL* Anatomical Automatic Labeling, *MNI* Montreal Neurological Institute

^*^P < 0.001, uncorrected, cluster size > 25 voxels

### Partial correlation analysis of FDG metabolism values and FOG-Q scores in differential brain regions between PD-FOG and PD-NFOG groups

**Fig. 3:**
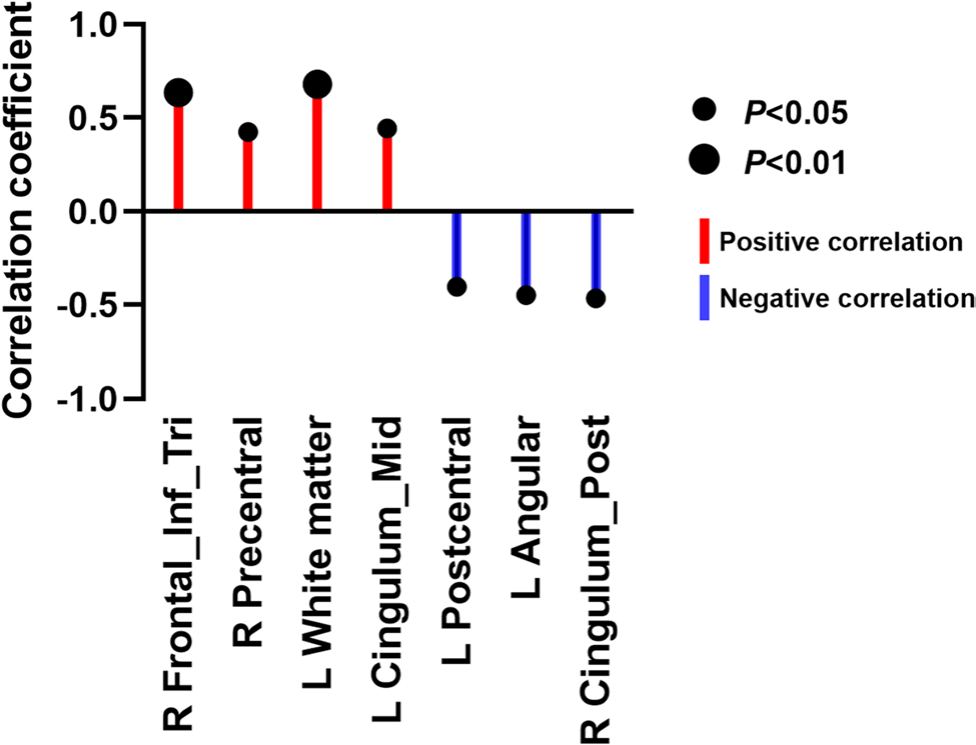
Partial correlation analysis between FDG metabolism values in differential brain regions and FOG-Q scores in the PD-FOG and PD-NFOG groups. Red circles represent positive correlations, blue circles represent negative correlations, and the circle size reflects the significance level (based on FDR-corrected p-values). Detailed correlation coefficients and FDR-corrected p-values are provided in Supplementary Table S2. Abbreviations: Frontal_Inf_Tri R, right inferior frontal gyrus (triangular part); Precentral R, right precentral gyrus; L Primary Somatosensory 1, left primary somatosensory cortex (area 1); Cingulum_Mid L, left mid-cingulate cortex; L Primary Somatosensory 2, left secondary somatosensory cortex (area 2); Angular L, left angular gyrus; Cingulum_Post R, right posterior cingulate cortex

The triangular part of the right inferior frontal gyrus (r = 0.636, FDR p < 0.001), the right precentral gyrus (r = 0.424, FDR p = 0.028), the left primary somatosensory cortex area 1 (r = 0.680, FDR p < 0.001), and the left middle cingulate gyrus (r = 0.444, FDR p = 0.020) were significantly positively correlated with FOG-Q scores. In contrast, the left secondary somatosensory cortex area 2 (r = –0.404, FDR p = 0.036), the left angular gyrus (r = –0.447, FDR p = 0.019), and the right posterior cingulate gyrus (r = –0.465, FDR p = 0.015) were significantly negatively correlated with FOG-Q scores. These findings are illustrated in Figure 3, while detailed correlation coefficients and FDR-corrected p-values are summarized in Supplementary Table S2. Collectively, the results reveal a complex network structure in which positively and negatively correlated regions were internally coordinated but exhibited antagonistic relationships across groups.

### The diagnostic efficacy of deep learning for PD and PD-FOG

**Fig. 4:**
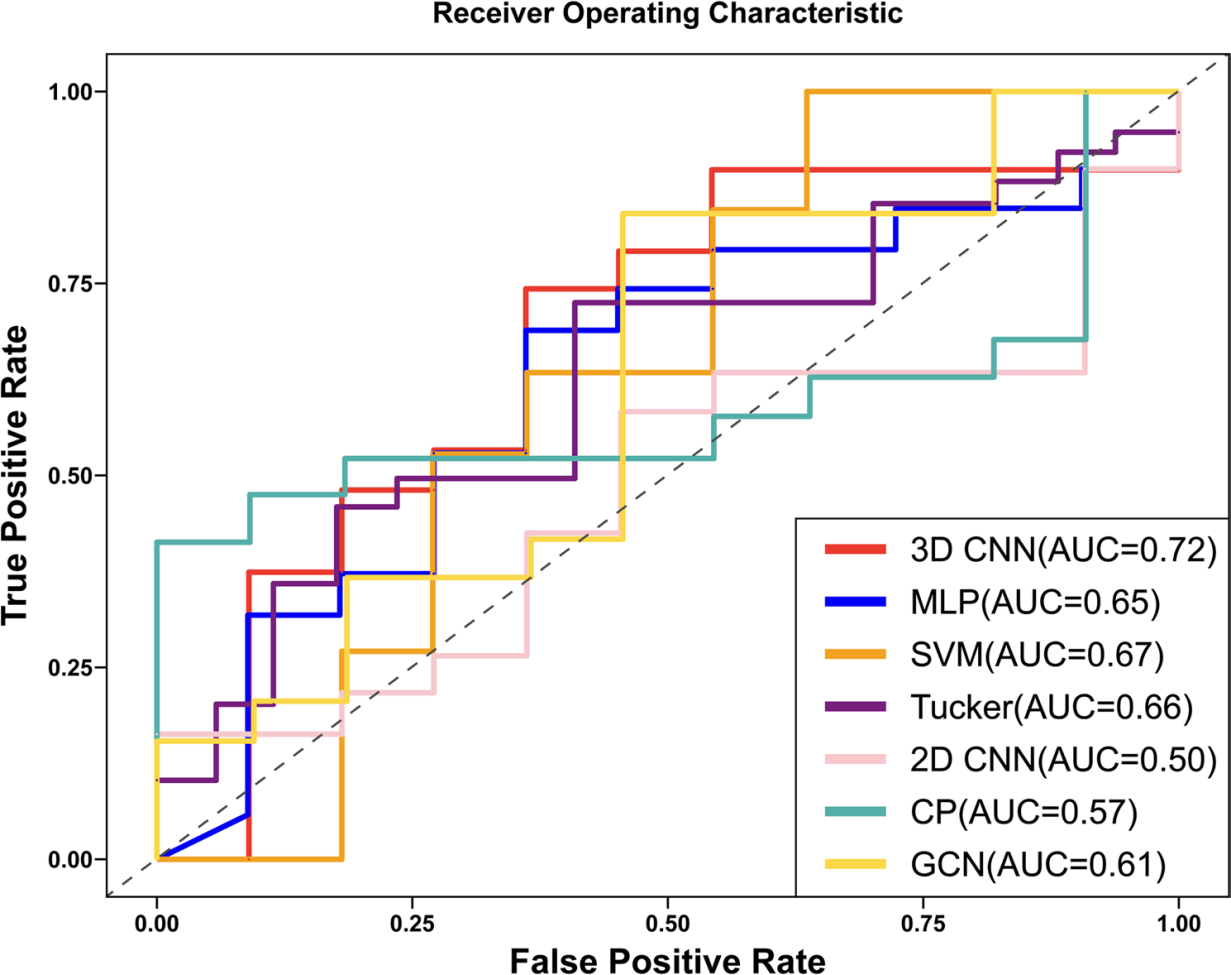
ROC curves of deep learning methods for PD-FOG diagnosis. All AUC values were recalculated to ensure ≥ 0.5 after correcting for potential label inversion in ROC computation. Abbreviations: MLP, Multi-Layer Perceptron; SVM, Support Vector Machine; GCN, Graph Convolutional Neural Network; 2D CNN, two-dimensional convolutional neural network; 3D CNN, three-dimensional convolutional neural network

We employed seven classifiers—MLP, SVM, GCN, Tucker, CP, 2D CNN, and 3D CNN—to establish models for distinguishing PD and PD-FOG. The sensitivity, specificity, accuracy, and AUC of each model for the tasks of PD diagnosis and PD-FOG diagnosis are shown in Table S1 and Table 3. Their corresponding ROC plots are presented in Figure S1 and Figure 4. To ensure consistent interpretation, all AUC values were recalculated to be ≥ 0.5 after correcting for potential label inversion in ROC computation.

For the PD diagnosis task, the results indicated that the designed 3D CNN model outperformed other diagnostic models in predicting PD, achieving higher levels of accuracy and recall, along with the best F1 score and AUC values. Specifically, the 3D CNN model achieved an accuracy of 90.09%, precision of 87.89%, recall of 98.84%, an AUC of 0.82, and an F1 score of 92.85 (Table S1; Fig. S1). The 2D CNN model achieved an accuracy of 78.50% with an AUC of 0.69 after correction, while other models such as MLP, SVM, GCN, Tucker, and CP yielded AUC values of 0.64–0.70.

For the PD-FOG diagnosis task, the 3D CNN model also demonstrated superior performance, with an accuracy of 95.40%, precision of 97.93%, recall of 95.68%, an AUC of 0.72, and an F1 score of 96.38 (Table 3; Fig. 4). This outperformance of the 3D CNN model was evident when compared to other models, including the MLP, which achieved accuracies of 87.43% for PD diagnosis and 85.05% for PD-FOG diagnosis; the SVM, with 69.56% accuracy for PD diagnosis and 75.86% for PD-FOG diagnosis; and the GCN, which showed 67.39% accuracy in PD diagnosis and 69.54% in PD-FOG diagnosis, with an AUC corrected to 0.61. Other models like Tucker and CP performed with accuracies of 71.73% and 62.06% for PD diagnosis, and 67.39% and 62.06% for PD-FOG diagnosis, with corrected AUC values of 0.66 and 0.57, respectively.

**Table 3:** Diagnostic results of deep learning methods for the classification of PD-FOG (mean ± SD)

Method (%)	Accuracy (%)	Precision (%)	Recall (%)	F1-score (%)	AUC
MLP	85.05 ± 8.00	88.39 ± 11.69	91.95 ± 12.08	88.79 ± 5.73	0.65
SVM	75.86 ± 42.79	79.31 ± 40.50	96.55 ± 18.24	75.86 ± 42.79	0.67
GCN	69.54 ± 27.35	4.59 ± 8.63	24.13 ± 42.79	7.63 ± 14.02	0.61
Tucker	67.39 ± 46.87	73.91 ± 43.91	93.47 ± 24.69	67.39 ± 46.87	0.66
CP	62.06 ± 48.52	79.31 ± 40.50	82.75 ± 37.77	62.06 ± 48.52	0.57
2D CNN	60.34 ± 9.19	60.91 ± 10.96	83.04 ± 16.44	68.24 ± 4.72	0.50
3D CNN	95.40 ± 7.44	97.93 ± 6.09	95.68 ± 9.44	96.38 ± 5.08	0.72

*Abbreviations*:* MLP* Multi-Layer Perceptron, *SVM* Support Vector Machine, *GCN* Graph Convolutional Neural Network, *Tucker* Tucker decomposition, *CP* Canonical Polyadic decomposition, *2D CNN* two-dimensional convolutional neural network, *3D CNN* three-dimensional convolutional neural network

### Performance of 3D CNN and traditional regression methods in evaluating FOG-Q scores

In regression analysis, Mean Squared Error (MSE), Root Mean Squared Error (RMSE), and Mean Absolute Error (MAE) are commonly used to quantify model performance. Smaller values indicate better predictive accuracy.

When comparing our 3D CNN with three traditional regression methods (Polynomial, DecisionTree, and RandomForest), the 3D CNN achieved the lowest MAE (4.44 ± 4.36) and RMSE (4.44 ± 5.31), and a competitive MSE (48.01 ± 96.82), demonstrating superior overall performance (Table 4). The Polynomial regression model showed comparable MSE (48.86 ± 74.92) but a larger MAE (5.82 ± 3.86) and RMSE (5.82 ± 3.86). The DecisionTree model performed the worst, with the largest MSE (103.86 ± 133.55), MAE (7.17 ± 7.23), and RMSE (7.17 ± 7.23). Although RandomForest achieved a lower MSE (42.64 ± 75.74) than the 3D CNN, its MAE (5.07 ± 4.10) and RMSE (5.07 ± 4.10) were still higher, indicating that the 3D CNN provided more balanced and stable predictions.

These findings highlight the effectiveness of the 3D CNN in accurately predicting FOG-Q scores, outperforming traditional machine learning approaches in terms of regression reliability and consistency.

**Table 4:** The deep learning method was used to diagnose the FOG-Q scores (mean ± SD)

Method	MSE	MAE	RMSE
3D CNN	48.01 ± 96.82	4.44 ± 4.36	4.44 ± 5.31
Polynomial	48.86 ± 74.92	5.82 ± 3.86	5.82 ± 3.86
DecisionTree	103.86 ± 133.55	7.17 ± 7.23	7.17 ± 7.23
RandomForest	42.64 ± 75.74	5.07 ± 4.10	5.07 ± 4.10

*Abbreviations*: *3D CNN* three-dimensional convolutional neural network, *Polynomial* polynomial regression, *DecisionTree* decision tree regression, *RandomForest* random forest regression

## Discussion

This study combined 18F-FDG PET imaging with deep learning techniques to explore the unique patterns of brain glucose metabolism in PD-FOG and to evaluate the performance of 3D CNN in the diagnosis of PD-FOG.

### Changes in glucose metabolism patterns in FOG

After excluding the interference of disease severity by partial correlation analysis, we found that, compared with the PD-NFOG group, the PD-FOG group exhibited significantly reduced glucose metabolism in areas such as left angular gyrus, left primary somatosensory area 2, and right posterior cingulate gyrus. These brain regions are closely related to motor planning, somatosensory processing, and motor execution. Dysfunction of the motor control network in FOG patients has been suggested in several studies [2, 11, 37]. Previous studies have shown that the posterior cingulate gyrus is a crucial connection point between cortical and subcortical motor circuits, and its decreased metabolism may reflect impaired motor coordination in PD-FOG patients [38]. Moreover, the reduced metabolism in the left primary somatosensory area 2 could be associated with diminished sensory-motor integration, which aligns with the gait instability and feedforward control deficits commonly reported by many FOG patients [39]. In contrast, the glucose metabolism in the PD-FOG group significantly increased in areas such as the right inferiortriangle of the frontal gyrus, the left first somatosensory cortex,the right anterior central gyrus, and the middle part of the left cingulate gyrus. Additionally, the increased metabolism in the precentral gyrus may be related to FOG patients' efforts to adjust motor planning and execution spontaneously [40]. Abnormal increases in glucose metabolism in the inferior frontal gyrus and middle cingulate cortex may be associated with compensatory mechanisms for gait control. Especially in the case of freezing and unsteady gait in Parkinson's disease, increased metabolism in these regions could be an adaptive response of the brain in response to movement disorders [41].

Compared to healthy controls, the PD-FOG group showed reduced metabolism in the medial superior frontal gyrus, putamen, and caudate nucleus while exhibiting increased metabolism in areas such as the insula, parietal lobe, and cerebellum. These findings suggest that FOG may involve not only alterations in the motor control network but also dysfunction within the cognitive-motor integration network [42]. Specifically, reduced metabolism in the medial superior frontal gyrus could be linked to impaired attention allocation in multitasking situations among FOG patients [43]. The decreased metabolism in the putamen and caudate nucleus further supports the possible role of basal ganglia–cortical loop dysfunction in the pathogenesis of FOG. Several studies have indicated that FOG patients exhibit higher failure rates in dual-task conditions, suggesting that cognitive load may significantly influence motor control. Meanwhile, the cerebellum may serve a compensatory role in PD patients by enhancing sensory feedback regulation to counterbalance deficits in cortical motor networks [44]. The overactivation of the cerebellum may also partially explain the excessive effort reported by many FOG patients during gait tasks [45]. In our study, increased parietal lobe and insula metabolism further supports the notion that FOG patients may rely on additional cognitive resources to perform even relatively simple gait tasks.

The complexity of metabolic changes in brain regions related to execution can be interpreted either as dysfunction of motor control networks or as adaptive compensatory responses of the brain to cope with motor symptoms such as freezing and instability of gait. The different directions of these metabolic changes (decreased metabolism in some regions and increased metabolism in others) reflect the complex adaptive responses in PD-FOG patients during the course of neurodegeneration. Moreover, the metabolic changes in these brain regions were significantly correlated with the severity of FOG.

Importantly, our findings are consistent with previously published FDG-PET and fMRI studies of FOG. For example, FDG-PET studies have frequently reported hypometabolism in the posterior cingulate cortex, basal ganglia, and angular gyrus—regions also identified as impaired in our PD-FOG patients [46]. Similarly, fMRI studies have shown disrupted functional connectivity involving the supplementary motor area and cingulate cortex, which parallels the hypermetabolism observed in these regions in our cohort [47, 48]. Such convergences across imaging modalities reinforce the robustness of our results and support the view that FOG may arise from dysfunction involving both motor control and cognitive-motor integration networks. These multimodal consistencies increase confidence in the reliability of our findings.

### Superior performance of 3D CNN in FOG diagnosis and severity assessment

In this study, the 3D CNN model showed higher performance compared with traditional methods in FOG classification, achieving 95.40% accuracy and 96.38% F1 score. This performance can be attributed to the ability of 3D CNNs to directly process voxel-based imaging data, making full use of the spatial and contextual information contained in PET scans [49]. While other machine learning approaches, such as SVMs, GCNs, or tensor decomposition methods, can also process voxel-based data [50], the unique advantage of 3D CNN lies in its ability to automatically learn hierarchical spatial features without relying on handcrafted features, thereby capturing more complex and distributed patterns of altered glucose metabolism in PD-FOG [51].

Our findings were consistent with prior studies. For example, Thi Khuyen Le et al. demonstrated that a 3D CNN could automatically diagnose neurodegenerative Parkinson’s syndrome and reduce diagnostic errors by 6% in inexperienced hospitals [16]. Similar strengths of 3D CNN have also been confirmed in studies of Alzheimer’s disease and multiple sclerosis [21, 52]. The powerful performance of 3D CNN was also due to its low demand for feature engineering and its adaptability to high-dimensional data. This study used 10-FCV to enhance the model’s robustness and avoid overfitting, which is widely applied in deep learning research with small sample datasets [53].

Although the 3D CNN performed well in our study, its interpretability remains limited. Recent advances in explainable deep learning, such as attention mechanisms and saliency maps, may provide strategies to improve transparency. For instance, generating saliency maps could help visually highlight the key brain regions that the 3D CNN model emphasizes during classification, thereby enhancing clinical interpretability.

### Practical applications and future directions

Imaging PD patients with FDG-PET may provide valuable insights into the metabolic networks underlying freezing of gait, which in turn may facilitate the development of targeted interventions and guide patient selection for clinical trials. Although FDG-PET is unlikely to be used for routine diagnostic screening, it may provide complementary information to standard clinical monitoring and help refine therapeutic strategies.

Future studies should address current gaps by enrolling larger and more diverse cohorts, adopting longitudinal and multi-center designs, and integrating multimodal imaging modalities such as MRI and fMRI. In addition, the application of explainable deep learning techniques, including attention mechanisms and saliency mapping, may improve interpretability and accelerate translation of these methods into clinical practice.

### Limitations

Several limitations should be acknowledged. First, the relatively small sample size may reduce generalizability and increase the risk of overfitting, despite the use of 10-fold cross-validation. Second, FDG-PET images were normalized using a global mean uptake method, which is semi-quantitative and does not provide absolute metabolic rates. Third, the cross-sectional design precludes causal inference regarding the relationship between metabolic alterations and FOG. Fourth, external validation with independent and multi-center datasets was not performed, which may limit the robustness and generalizability of our findings. Fifth, multimodal imaging (such as MRI or fMRI) and detailed clinical rating scales were not incorporated for cross-validation, which could have provided more comprehensive insights. Finally, although the 3D CNN model showed encouraging performance, its interpretability and clinical utility remain to be further validated in prospective studies.

## Conclusion

This study combined 18F-FDG PET imaging and 3D CNN deep learning to identify significant metabolic changes in specific brain regions of PD-FOG patients. These findings may help to elucidate the brain networks associated with FOG, providing potential insights into underlying mechanisms and therapeutic targets. The 3D CNN achieved an accuracy of 95.40%, suggesting its potential as an auxiliary diagnostic tool for freezing of gait in Parkinson’s disease and supporting the clinical applicability of artificial intelligence in neurodegenerative disorders.

## Supplementary Information

Supplementary Material 1

## Data Availability

The data and materials used along the current study are available from the corresponding author on reasonable request.

## Abbreviations

PD-FOG: Parkinson’s disease patients with freezing of gait
PD-NFOG: Parkinson’s disease patients without freezing of gait
HC: healthy controls
L: Left
R: Right
