# Supplementary material for: Characteristics of brain glucose metabolism in Parkinson’s disease patients with freezing of gait: a study based on 18F-FDG PET imaging and deep learning
Source: BMC Neurol. 2025 Oct 31;25:446. doi: 10.1186/s12883-025-04468-y (PMC12577301; doi:10.1186/s12883-025-04468-y)
Supplement: Supplementary file 1 — Supplementary Material 1 [file 12883_2025_4468_MOESM1_ESM.docx]

**SUPPLEMENTAL MATERIAL**

**Table S1 Diagnostic results of deep learning methods for PD classification (mean ± SD).**

| Method | Accuracy (%) | Precision | Recall | F1-score | AUC |
| --- | --- | --- | --- | --- | --- |
| MLP | 87.43 ± 6.79 | 88.97 ± 8.25 | 97.20 ± 5.66 | 92.48 ± 3.82 | 0.64 |
| SVM | 69.56 ± 46.01 | 78.26 ± 41.24 | 91.30 ± 28.17 | 69.56 ± 46.01 | 0.63 |
| GCN | 67.39 ± 46.87 | 76.08 ± 42.65 | 91.30 ± 28.17 | 67.39 ± 46.87 | 0.64 |
| Tucker | 71.73 ± 41.24 | 78.26 ± 41.24 | 93.47 ± 24.69 | 71.73 ± 45.02 | 0.70 |
| CP | 60.86 ± 48.80 | 76.08 ± 42.65 | 84.78 ± 35.91 | 60.86 ± 48.80 | 0.66 |
| 2D CNN | 78.50 ± 2.74 | 78.74 ± 3.89 | 99.68 ± 2.08 | 87.89 ± 1.50 | 0.69 |
| 3D CNN | 90.09 ± 4.76 | 87.89 ± 6.24 | 98.84 ± 4.40 | 92.85 ± 3.63 | 0.82 |

Abbreviations: MLP, Multi-Layer Perceptron; SVM, Support Vector Machine; GCN, Graph Convolutional Neural Network; Tucker, Tucker decomposition; CP, Canonical Polyadic decomposition; 2D CNN, two-dimensional convolutional neural network; 3D CNN, three-dimensional convolutional neural network.

**Table S2. Partial correlation analysis between FDG metabolism values in differential brain regions and FOG-Q (PD-FOG vs PD-NFOG)**

| Brain region | Correlation coefficient (r) | FDR-corrected *p*-value |
| --- | --- | --- |
| Right inferior frontal gyrus, triangular part | 0.636 | <0.001 |
| Right precentral gyrus | 0.424 | 0.028 |
| Left primary somatosensory cortex (area 1) | 0.68 | <0.001 |
| Left middle cingulate gyrus | 0.444 | 0.020 |
| Left secondary somatosensory cortex (area 2) | -0.404 | 0.036 |
| Left angular gyrus | -0.447 | 0.019 |
| Right posterior cingulate gyrus | -0.465 | 0.015 |


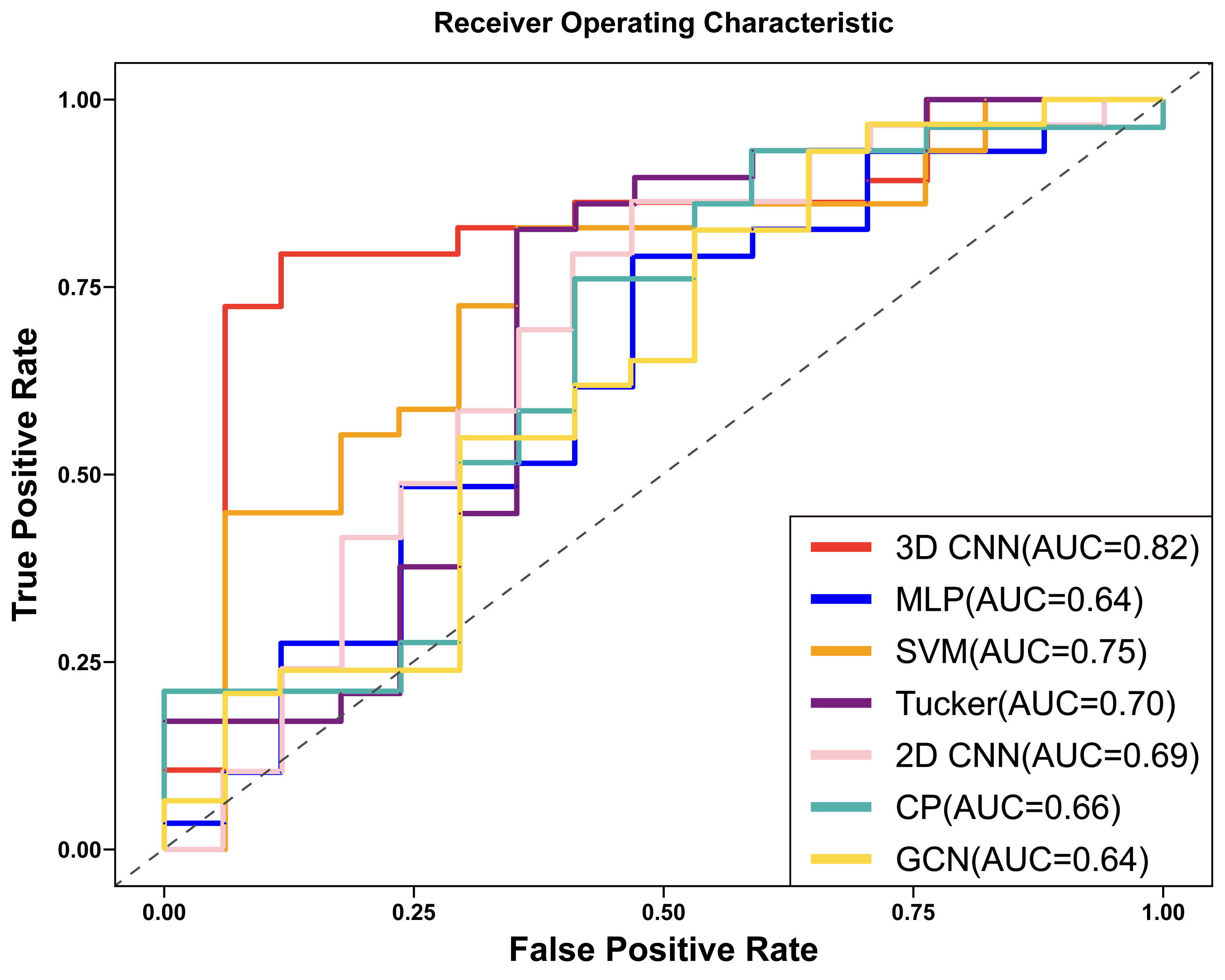


Figure S1. ROC curves of deep learning methods for PD diagnosis. All AUC values were recalculated to ensure ≥ 0.5 after correcting for potential label inversion in ROC computation. Abbreviations: MLP, Multi-Layer Perceptron; SVM, Support Vector Machine; GCN, Graph Convolutional Neural Network; 2D CNN, two-dimensional convolutional neural network; 3D CNN, three-dimensional convolutional neural network.

**Deep learning model code**

import math

import os

import random

import warnings

import torch

from matplotlib import pyplot as plt

from scipy.io import loadmat

from sklearn.metrics import precision_score, recall_score, f1_score, roc_auc_score, accuracy_score, confusion_matrix

from sklearn.model_selection import KFold

from torch import nn

from torch.utils.data import SubsetRandomSampler, DataLoader, Dataset

import torch.nn.functional as F

import numpy as np

from matplotlib import pyplot as plt

from scipy.io import loadmat

from torch.utils.data import Dataset

import tensorly as tl

from tensorly.decomposition import parafac

import tensorly as tl

from tensorly.decomposition import tucker

from sklearn.metrics import roc_curve, auc

import matplotlib.pyplot as plt

warnings.filterwarnings("ignore")

os.environ['KMP_DUPLICATE_LIB_OK'] = 'TRUE'

DEVICE = torch.device("cuda:0" if torch.cuda.is_available() else "cpu")

############################################### seed = 7

torch.manual_seed(seed)

torch.cuda.manual_seed(seed)

torch.cuda.manual_seed_all(seed)

np.random.seed(seed)

random.seed(seed)

############################################### ####################################

def stest(model, datasets_test, num_win, mode=None):

eval_loss = 0

eval_acc = 0

pre_all = []

labels_all = []

pro_all = []

score_all = []

model.eval()

with torch.no_grad():

for tg, label in datasets_test:

tg, label = tg.to(DEVICE), label.to(DEVICE)

tg = tg.float()

tg = tg.unsqueeze(1)

label = label.long()

# print(label.shape)

outs = model(tg)

losss = F.nll_loss(outs, label)

eval_loss += float(losss)

gailv, pred = outs.max(1)

num_correct = (pred == label).sum()

acc = int(num_correct) / tg.shape[0]

eval_acc += acc

pre = pred.cpu().detach().numpy()

pre_all.extend(pre)

label_true = label.cpu().detach().numpy()

labels_all.extend(label_true)

pro_all.extend(outs[:, 1].cpu().detach().numpy())

if mode is True:

score_all.extend(torch.exp(outs[:, 1]).cpu().detach().numpy())

# tn, fp, fn, tp = confusion_matrix(labels_all, pre_all).ravel()

# sensitivity = tp / (tp + fn)

# specificity = tn / (tn + fp)

eval_acc_epoch = accuracy_score(labels_all, pre_all)

precision = tp/（tp + fp)

precision = precision_score(labels_all, pre_all)

recall = recall_score(labels_all, pre_all)

f1 = f1_score(labels_all, pre_all)

# print(len(labels_all))

# print(len(pre_all))

# my_auc = roc_auc_score(labels_all, pro_all)

return eval_loss, eval_acc, eval_acc_epoch, precision, recall, f1, pre_all, labels_all, pro_all, score_all

def MaxMinNormalization(x, Max, Min):

x = (x - Min) / (Max - Min)

return x

class PD(Dataset):

def __init__(self):

super(PD, self).__init__()

self.feas = fdata

self.label = labels

def __getitem__(self, item):

fea = self.feas[item]

label = self.label[item]

return fea, label

def __len__(self):

return self.feas.shape[0]

class MLP(nn.Module):

def __init__(self, ):

super(MLP, self).__init__()

self.f1 = nn.Flatten()

self.l1 = nn.Linear(95 * 79 * 79, 128)

self.bn1 = nn.BatchNorm1d(128)

self.d1 = nn.Dropout(p=0.6)

self.l2 = nn.Linear(128, 64)

self.bn2 = nn.BatchNorm1d(64)

self.d2 = nn.Dropout(p=0.6)

self.l3 = nn.Linear(64, 2)

self.logs = nn.LogSoftmax(dim=1)

def forward(self, x):

out = self.f1(x)

# print(out.shape)

out = self.d1(self.bn1(self.l1(out)))

out = self.d2(self.bn2(self.l2(out)))

out = self.logs(self.l3(out + 1e-8))

return out

class Simple3DCNN(nn.Module):

def __init__(self):

super(Simple3DCNN, self).__init__()

self.conv1 = nn.Conv3d(in_channels=1, out_channels=16, kernel_size=3, stride=1, padding=1)

self.pool1 = nn.MaxPool3d(kernel_size=2, stride=2, padding=0)

self.conv2 = nn.Conv3d(in_channels=16, out_channels=32, kernel_size=3, stride=1, padding=1)

self.pool2 = nn.MaxPool3d(kernel_size=2, stride=2, padding=0)

self.conv3 = nn.Conv3d(in_channels=32, out_channels=64, kernel_size=3, stride=1, padding=1)

self.pool3 = nn.MaxPool3d(kernel_size=2, stride=2, padding=0)

self.flattened_size = 64 * 11 * 9 * 9

self.fc1 = nn.Linear(self.flattened_size, 128)

self.fc2 = nn.Linear(128, 2)

self.logs = nn.LogSoftmax(dim=1)

def forward(self, x):

x = self.pool1(F.relu(self.conv1(x)))

x = self.pool2(F.relu(self.conv2(x)))

x = self.pool3(F.relu(self.conv3(x)))

x = x.view(-1, self.flattened_size) ([4, 57024])

x = F.relu(self.fc1(x)) ([4, 128])

x = self.fc2(x)

x = self.logs(x + 1e-8)

return x

def tucker_decomposition(data, rank):

tensor = tl.tensor(data)

core, factors = tucker(tensor, rank=rank)

return core, factors

wd = 1e-3

log = open('logs_3d_ill.txt', mode='a', encoding='utf-8')

for he_dim in [4]:

for yu in [0.3]:

for drop in [0, 1e-4,2e-3,1e-3]:

for lr1 in [3e-4, 5e-4, 7e-4, 1e-3, 3e-3, 5e-3]:

m = loadmat('.\PD_PET.mat') # fmri

keysm = list(m.keys())

fdata = m[keysm[3]]

labels = m[keysm[4]][0]

#

# # temp_data = []

# temp_label = []

# for i in range(fdata.shape[0]):

# if labels[i] == 1:

# temp_data.append(fdata[i])

# temp_label.append(1)

# if labels[i] == 2:

# temp_data.append(fdata[i])

# temp_label.append(0)

#

# fdata = np.array(temp_data)

# labels = np.array(temp_label)

# print(fdata.shape)

# print("lable:", labels)

for i in range(fdata.shape[0]):

for j in range(fdata.shape[1]):

max_t = np.max(fdata[i, j, :, :])

min_t = np.min(fdata[i, j, :, :])

fdata[i, j, :, :] = MaxMinNormalization(fdata[i, j, :, :], max_t, min_t)

for i in range(labels.shape[0]):

if labels[i] == 2:

labels[i] = 1

print(fdata.shape)

print("lable:", labels)

index = [i for i in range(fdata.shape[0])]

np.random.shuffle(index)

fdata = fdata[index]

labels = labels[index]

num_win = 2

n_class = 2

avg_acc = 0

avg_spe = 0

avg_recall = 0

avg_f1 = 0

avg_auc = 0

avg_sens = 0

avg_spec = 0

pre_ten = []

label_ten = []

pro_ten = []

test_acc = []

test_pre = []

test_recall = []

test_f1 = []

test_auc = []

test_sens = []

test_spec = []

dataset = PD()

k = 10

i = 0

train_ratio = 0.8

# beta = 0.5

KF = KFold(n_splits=k, shuffle=True, random_state=7)

y_true = []

y_score = []

for train_idx, test_idx in KF.split(dataset):

train_size = int(train_ratio * len(train_idx))

valid_size = len(train_idx) - train_size

train_indices, valid_indices = train_idx[:train_size], train_idx[train_size:]

datasets_train = DataLoader(dataset, batch_size=16, shuffle=False,

sampler=SubsetRandomSampler(train_indices))

datasets_valid = DataLoader(dataset, batch_size=16, shuffle=False,

sampler=SubsetRandomSampler(valid_indices))

datasets_test = DataLoader(dataset, batch_size=1, shuffle=False,

sampler=SubsetRandomSampler(test_idx))

min_loss = 1e10

losses = []

acces = []

eval_losses = []

eval_acces = []

patience = 0

patiences = 100

min_acc = 0

pre_gd = 0

recall_gd = 0

f1_gd = 0

auc_gd = 0

sens_gd = 0

spec_gd = 0

labels_all_gd = 0

pro_all_gd = 0

model = Simple3DCNN()

model.to(DEVICE)

optimizer = torch.optim.Adam(model.parameters(), lr=lr1, weight_decay=drop)

# scheduler = torch.optim.lr_scheduler.StepLR(optimizer, step_size=10, gamma=0.3)

for e in range(300):

model.train()

train_loss = 0

train_acc = 0

pre_all_train = []

labels_all_train = []

model.train()

for tg, label in datasets_train:

tg, label = tg.to(DEVICE), label.to(DEVICE)

tg = tg.float()

# 1111111

tg = tg.unsqueeze(1)

label = label.long()

# print(label.shape)

out = model(tg)

loss_c = F.nll_loss(out, label)

optimizer.zero_grad()

loss_c.backward()

torch.nn.utils.clip_grad_norm_(model.parameters(), 1)

optimizer.step()

train_loss += float(loss_c)

_, pred = out.max(1)

pre = pred.cpu().detach().numpy()

pre_all_train.extend(pre)

label_true = label.cpu().detach().numpy()

labels_all_train.extend(label_true)

# scheduler.step()

losses.append(train_loss / len(datasets_train))

acces.append(train_acc / len(datasets_train))

train_acc = accuracy_score(labels_all_train, pre_all_train)

eval_loss, eval_acc, eval_acc_epoch, precision, recall, f1, pre_all, labels_all, pro_all, score_all = stest(

model, datasets_valid, num_win)

if eval_acc_epoch > min_acc:

torch.save(model.state_dict(), './latest' + str(i) + '.pth')

print("Model saved at epoch{}".format(e))

min_acc = eval_acc_epoch

pre_gd = precision

recall_gd = recall

f1_gd = f1

labels_all_gd = labels_all

pro_all_gd = pro_all

patience = 0

else:

patience += 1

if patience > patiences:

break

eval_losses.append(eval_loss / len(datasets_test))

eval_acces.append(eval_acc / len(datasets_test))

print(

'i:{},epoch: {}, Train Loss: {:.6f}, Train Acc: {:.6f}, Eval Loss: {:.6f}, Eval Acc: {:.6f},precision : {'

':.6f},recall : {:.6f},f1 : {:.6f}, '

.format(i, e, train_loss / len(datasets_train), train_acc,

eval_loss / len(datasets_test),

eval_acc_epoch,

precision, recall, f1))

model_test = Simple3DCNN()

model_test = model_test.to(DEVICE)

model_test.load_state_dict(torch.load('./latest' + str(i) + '.pth')) # 84.3750

print("-----我要开始测试啦-------")

eval_loss, eval_acc, eval_acc_epoch, precision, recall, f1, pre_all, labels_all, pro_all, score_all = stest(

model_test, datasets_test, num_win, mode=True)

y_true.append(labels_all)

y_score.append(score_all)

test_acc.append(min_acc)

test_pre.append(pre_gd)

test_recall.append(recall_gd)

test_f1.append(f1_gd)

test_auc.append(auc_gd)

test_sens.append(sens_gd)

test_spec.append(spec_gd)

label_ten.extend(labels_all_gd)

pro_ten.extend(pro_all_gd)

i = i + 1

print("num_win", num_win, 'he_dim', he_dim, 'yu', yu, 'drop', drop, 'lr', lr1, 'wd', wd,

"test_acc",

test_acc, file=log)

print("num_win", num_win, 'he_dim', he_dim, 'yu', yu, 'drop', drop, 'lr', lr1, 'wd', wd,

"test_pre",

test_pre, file=log)

print("num_win", num_win, 'he_dim', he_dim, 'yu', yu, 'drop', drop, 'lr', lr1, 'wd', wd,

"test_recall",

test_recall, file=log)

print("num_win", num_win, 'he_dim', he_dim, 'yu', yu, 'drop', drop, 'lr', lr1, 'wd', wd,

"test_f1",

test_f1, file=log)

print("num_win", num_win, 'he_dim', he_dim, 'yu', yu, 'drop', drop, 'lr', lr1, 'wd', wd,

"test_auc",

test_auc, file=log)

print("num_win", num_win, 'he_dim', he_dim, 'yu', yu, 'drop', drop, 'lr', lr1, 'wd', wd,

"test_sens",

test_sens, file=log)

print("num_win", num_win, 'he_dim', he_dim, 'yu', yu, 'drop', drop, 'lr', lr1, 'wd', wd,

"test_spec",

test_spec, file=log)

avg_acc = sum(test_acc) / k

avg_pre = sum(test_pre) / k

avg_recall = sum(test_recall) / k

avg_f1 = sum(test_f1) / k

fpr, tpr, thresholds = roc_curve(y_true, y_score)

roc_auc = auc(fpr, tpr)

plt.figure()

plt.plot(fpr, tpr, color='darkorange', lw=2, label='ROC curve (area = %0.2f)' % roc_auc)

plt.plot([0, 1], [0, 1], color='navy', lw=2, linestyle='--')

plt.xlim([0.0, 1.0])

plt.ylim([0.0, 1.05])

plt.xlabel('False Positive Rate')

plt.ylabel('True Positive Rate')

plt.title('Receiver Operating Characteristic')

plt.legend(loc="lower right")

plt.savefig('./roc_3d_ill/roc_lr{}_drop{}.png'.format(lr1, drop))

print("---------roc_lr{}_drop{} roc--------".format(lr1, drop))

print("auc", roc_auc, "y_true", y_true, "y_score", y_score)

print("*****************************************************", file=log)

print("lr", lr1, "drop", drop, "auc", roc_auc, "y_true", y_true, "y_score", y_score,file=log)

print("num_win", num_win, 'he_dim', he_dim, 'yu', yu, 'drop', drop, 'lr', lr1, 'wd', wd,

'acc', avg_acc,

file=log)

print("num_win", num_win, 'he_dim', he_dim, 'yu', yu, 'drop', drop, 'lr', lr1, 'wd', wd,

'pre', avg_pre,

file=log)

print("num_win", num_win, 'he_dim', he_dim, 'yu', yu, 'drop', drop, 'lr', lr1, 'wd', wd,

'recall',

avg_recall, file=log)

print("num_win", num_win, 'he_dim', he_dim, 'yu', yu, 'drop', drop, 'lr', lr1, 'wd', wd,

'f1', avg_f1,

file=log)

print("num_win", num_win, 'he_dim', he_dim, 'yu', yu, 'drop', drop, 'lr', lr1, 'wd', wd,

'auc', auc,

file=log)

# print("num_win", num_win, 'he_dim', he_dim, 'yu', yu, 'drop', drop, 'lr', lr1, 'wd', wd,

# 'auc', avg_auc,

# file=log)

# print("num_win", num_win, 'he_dim', he_dim, 'yu', yu, 'drop', drop, 'lr', lr1, 'wd', wd,

# "sensitivity",

# avg_sens, file=log)

# print("num_win", num_win, 'he_dim', he_dim, 'yu', yu, 'drop', drop, 'lr', lr1, 'wd', wd,

# "specificity",

# avg_spec, file=log)

acc_std = np.sqrt(np.var(test_acc))

pre_std = np.sqrt(np.var(test_pre))

recall_std = np.sqrt(np.var(test_recall))

f1_std = np.sqrt(np.var(test_f1))

# auc_std = np.sqrt(np.var(test_auc))

# sens_std = np.sqrt(np.var(test_sens))

# spec_std = np.sqrt(np.var(test_spec))

print("*****************************************************", file=log)

print("num_win", num_win, 'he_dim', he_dim, 'yu', yu, 'drop', drop, 'lr', lr1, 'wd', wd,

"acc_std",

acc_std, file=log)

print("num_win", num_win, 'he_dim', he_dim, 'yu', yu, 'drop', drop, 'lr', lr1, 'wd', wd,

"pre_std",

pre_std, file=log)

print("num_win", num_win, 'he_dim', he_dim, 'yu', yu, 'drop', drop, 'lr', lr1, 'wd', wd,

"recall_std",

recall_std, file=log)

print("num_win", num_win, 'he_dim', he_dim, 'yu', yu, 'drop', drop, 'lr', lr1, 'wd', wd,

"f1_std",

f1_std, file=log)

# print("num_win", num_win, 'he_dim', he_dim, 'yu', yu, 'drop', drop, 'lr', lr1, 'wd', wd,

# "auc_std",

# auc_std, file=log)

# print("num_win", num_win, 'he_dim', he_dim, 'yu', yu, 'drop', drop, 'lr', lr1, 'wd', wd,

# "sens_std",

# sens_std, file=log)

# print("num_win", num_win, 'he_dim', he_dim, 'yu', yu, 'drop', drop, 'lr', lr1, 'wd', wd,

# "spec_std",

# spec_std, file=log)

print("*****************************************************", file=log)

print("num_win", num_win, 'he_dim', he_dim, 'yu', yu, 'drop', drop, 'lr', lr1, 'wd', wd,

label_ten,

file=log)

print("num_win", num_win, 'he_dim', he_dim, 'yu', yu, 'drop', drop, 'lr', lr1, 'wd', wd,

pro_ten,

file=log)

print("*****************************************************", file=log)
